# An Insight into *Cotton Leaf Curl Multan Betasatellite*, the Most Important Component of Cotton Leaf Curl Disease Complex

**DOI:** 10.3390/v9100280

**Published:** 2017-09-29

**Authors:** Muhammad Zubair, Syed Shan-e-Ali Zaidi, Sara Shakir, Imran Amin, Shahid Mansoor

**Affiliations:** 1 National Institute for Biotechnology and Genetic Engineering, 38000 Faisalabad, Pakistan; zubair.nibge@gmail.com (M.Z.); shan.e.ali@outlook.com (S.S.Z.); shakir.sara@yahoo.com (S.S.); imranamin1@yahoo.com (I.A.); 2 Pakistan Institute of Engineering and Applied Sciences, Nilore, 45650 Islamabad, Pakistan; 3 AgroBioChem Department, Gembloux Agro-Bio Tech, University of Liège, 5030 Gembloux, Belgium; 4 Boyce Thompson Institute, 533 Tower Rd, Ithaca, NY 14853, USA

**Keywords:** Cotton leaf curl Multan betasatellite, βC1, begomovirus, leaf curl disease, Gossypium

## Abstract

Cotton leaf curl disease (CLCuD) is one of the most economically important diseases and is a constraint to cotton production in major producers, Pakistan and India. CLCuD is caused by monopartite plant viruses belonging to the family *Geminiviridae* (genus *Begomovirus*), in association with an essential, disease-specific satellite, *Cotton leaf curl Multan betasatellite* (CLCuMuB) belonging to a newly-established family *Tolecusatellitidae* (genus *Betasatellite*). CLCuMuB has a small genome (ca. 1350 nt) with a satellite conserved region, an adenine-rich region and a single gene that encodes for a multifunctional βC1 protein. CLCuMuB βC1 protein has a major role in pathogenicity and symptom determination, and alters several host cellular functions like autophagy, ubiquitination, and suppression of gene silencing, to assist CLCuD infectivity. Efficient *trans*-replication ability of CLCuMuB with several monopartite and bipartite begomoviruses, is also associated with the rapid evolution and spread of CLCuMuB. In this article we comprehensively reviewed the role of CLCuMuB in CLCuD, focusing on the βC1 functions and its interactions with host proteins.

## 1. Introduction−Begomoviruses and Betasatellites

**Figure 1:**
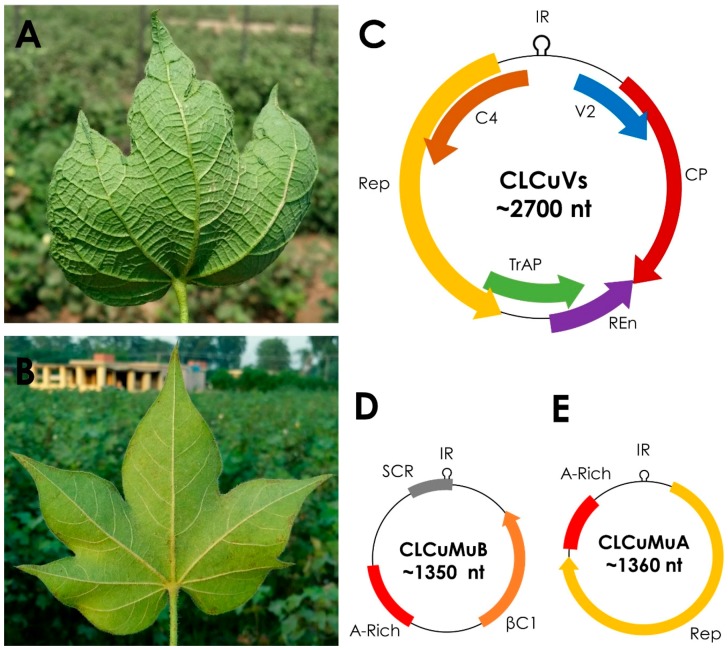
Symptoms of leaf curl disease on cotton and genome organization of the cotton leaf curl disease complex. (**A**) Cotton leaf curl disease (CLCuD) infected *Gossypium hirsutum* plant’s leaf in a cotton field in Punjab, Pakistan. Typical begomovirus-betasatellite disease complex symptoms like leaf curling, small vein thickening, large vein thickening, and cup shaped leaf, are visible on the lower side of infected leaf, where in panel (**B**) a healthy cotton leaf is free from such symptoms. (**C**) Genome organization of cotton leaf curl disease associated begomoviruses (CLCuVs) having genome of ca. 2700 nucleotides (nt) with four genes in complementary sense orientation encoding for replication associated protein (Rep), transcriptional activation protein (TrAP), replication enhancer protein (REn) and C4 protein; and two genes in virion sense orientation encoding for coat protein (CP) and V2 protein. (**D**) *Cotton leaf curl Multan betasatellite* (CLCuMuB) has a genome of ca. 1350 nt and encodes for a single βC1 protein. Non-coding region of CLCuMuB contains satellite conserved region (SCR) and adenine-rich (A-rich) region. (**E**) Cotton leaf curl Multan alphasatellite (CLCuMuA) has a genome of ca. 1370 nt and encodes for Rep. A-rich region is also present in CLCuMuA.

Cotton leaf curl disease (CLCuD) causes major yield losses and is a threat to cotton production in Pakistan and India [1,2]. CLCuD is caused by single-stranded DNA (ssDNA) plant viruses belonging to the genus *Begomovirus* (family *Geminiviridae*) [3,4]. The family *Geminiviridae* is classified into nine genera, *Becurtovirus, Begomovirus, Capulavirus, Curtovirus, Mastrevirus, Eragrovirus, Grablovirus, Topocuvirus*, and *Turncurtovirus* [5,6]. *Begomovirus* is the largest genus in family *Geminiviridae* that consists of more than 320 species [5] and are transmitted by insect vector whitefly (*Bemisia tabaci*) [7,8]. CLCuD is characterized by typical begomovirus disease symptoms like leaf curling, small vein thickening, large vein thickening, small leaf like enations under leaf and growth stunting (Figure 1A), leading to overall reduced crop production. A CLCuD free cotton plant, on the other hand, is free from such symptoms (Figure 1B). Phylogenetically, begomoviruses are classified into two main groups, the Old World (OW) and the New World (NW) [9,10]. Based on their genome organization, begomoviruses are either bipartite (with two genomic component known as DNA-A and DNA-B) or monopartite (with single genomic component that resembles DNA-A of bipartite begomoviruses) [11,12]. The DNA-A component encodes for AV1 or coat protein (CP) and AV2 or pre-coat protein in the virion-sense orientation, and replication initiation protein (Rep), replication enhancer protein (REn), transcriptional activator protein (TrAP), and the C4 protein in the complementary-sense orientation (Figure 1C). The DNA-B component encodes for nuclear shuttle protein (NSP) and movement protein (MP) [13]. The reading frames of both DNA-A and DNA-B are separated into two clusters by an intergenic region. The DNA-A and DNA-B components of bipartite begomoviruses share a common region (CR) within intergenic region which contains conserved sequences between these components and a hairpin structure with a nonanucleotide (TAATATT/AC) sequence in it, which serves as origin of virion-strand DNA replication [14]. Begomoviruses in the OW are frequently associated with helper single-stranded DNA satellite molecules alphasatellites, betasatellites [15], and newly-characterized deltasatellites [16,17]. Alphasatellites encode for a single Rep protein (Figure 1E) whereas betasatellites encode for an important pathogenicity and symptom determinant βC1 protein (Figure 1D) [18]. It must be noted here that recently the family *Tolecusatellitidae* has been established to encompass these single-stranded DNA satellites known as betasatellites (genus *Betasatellite*) and related satellites (genus *Deltasatellite*) [19]. For consistency and accuracy the latest nomenclature has been used for betasatellites in this manuscript, which describes betasatellites as independent species in the genus *Betasatellite* [19].

The genome of betasatellites is about half the size of their helper *begomovirus* (ca. 1350 nucleotides), shares no sequence homology with the helper virus, except the nonanucleotide region within the stem-loop structure. The genome of betasatellite harbors a single *βC1* gene in the complementary-sense orientation, an adenine rich (A-rich) region and a satellite conserved region (SCR). The A-rich region is ca. 160–200 nt long with ca. 57–65% adenine contents; it is likely involved in increasing the genome size and helps in efficient encapsidation and systemic movement of betasatellites [20]. The A-rich region appears to have minor role in regulation of *βC1* gene promoter [21] and also assists replication of complementary-sense strand DNA molecules [22]. Like the typical satellite molecules, betasatellites depend on their helper virus for cell-to-cell movement, systemic spread throughout the host, replication and transmission through insect vector [15]. Significant work has been done on understanding of replication, transmission, interaction and pathogenicity of betasatellites, and it has also been comprehensively reviewed [15,23]. However, in this article we focus only on betasatellite associated with CLCuD, i.e., *Cotton leaf curl Multan betasatellite* (CLCuMuB).

## 2. Cotton Leaf Curl Virus Disease

Since the early 1990s, there have been two major epidemics of CLCuD affecting cotton in Pakistan [24]. The first epidemic of CLCuD during the 1990s was associated with distinct monopartite begomoviruses–*Cotton leaf curl Kokhran virus* (CLCuKoV), *Cotton leaf curl Multan virus* (CLCuMuV), *Cotton leaf curl Alabad virus* (CLCuAlV), and *Papaya leaf curl virus* (PaLCuV) [25,26]; and a single “Multan” strain of CLCuMuB (CLCuMuB^Mul^) [22,27,28]. The losses of the first CLCuD epidemic were overcome in Pakistan by introduction of CLCuD resistant varieties, developed through conventional breeding [29]. In the second CLCuD epidemic during the early 2000s, predominantly a single begomovirus known as *Cotton leaf curl Kokhran virus* Burewala (CLCuKoV-Bur; also previously known as *Cotton leaf curl Burewala virus* CLCuBuV) along with the recombinant “Burewala” strain of CLCuMuB (CLCuMuB^Bur^), was associated with CLCuD complex in Pakistan and India [30,31,32]. CLCuKoV-Bur is a recombinant virus containing sequences derived from CLCuKoV in the virion-sense orientation and CLCuMuV in the complementary-sense orientation [33], and encodes a truncated TrAP of 35 amino acids (aa) [30,34]. Both CLCuKoV and CLCuMuV were dominant in CLCuD-infected cotton during the first disease epidemic [28,30].

CLCuD complex is in the state of continuous change, evolving by process of mutations, recombination and component capture to overcome CLCuD resistance in resistant varieties [1]. During the screening of cotton breeding lines that were previously tolerant and later became susceptible to CLCuD, CLCuKoV-Bur with a longer but still truncated *TrAP* gene was identified [35]. This might suggest that more pathogenic CLCuD begomoviruses, having a full-length TrAP, could return to cotton if the earlier resistance is not maintained [35]. Multiple begomoviruses associated with the first CLCuD epidemic such as CLCuMuV, CLCuKoV, and CLCuAlV, all having full length TrAP, have also been identified from cultivated cotton, associated with a new strain of CLCuMuB, Vehari strain (CLCuMuB^Veh^) [36]. *Cotton leaf curl Gezira virus* (CLCuGV), that causes CLCuD in Africa, has been identified in association with CLCuMuB in cotton from Southern Pakistan [37]. Recently, a bipartite begomovirus *Tomato leaf curl New Delhi virus* (ToLCNDV) has been found frequently associated with CLCuD infected cotton in Pakistan [4,38]. These studies indicate that the nature of CLCuD complex is changing over the time and might lead to the next epidemic of CLCuD in the OW [2].

## 3. Functions of βC1 and Interaction with Host Proteins

CLCuD in Pakistan is caused by a single betasatellite, CLCuMuB, in association with at least six begomovirus species. In the post-resistance-breaking era, CLCuMuB, associated with resistance breaking strain CLCuKoV-Bur, was also recombinant, containing a small fragment of ca. 95 nucleotides within the SCR derived from *Tomato leaf curl betasatellite* [39]. CLCuMuB is required by the helper virus for the induction of specific leaf curl disease symptoms [40] where βC1 of CLCuMuB is pathogenicity determinant and induces disease symptoms when expressed through the *Potato Virus X* (PVX) vector [41]. CLCuMuB βC1 might play an important role in intracellular transport by co-localizing with the endoplasmic reticulum [42]; additionally CLCuMuB-βC1 also localizes at the cell periphery in association with punctate bodies, around and within the cell nucleus [42]. Studies on *B. tabaci*-mediated transmission of CLCuMuB suggested that CLCuMuB DNA is encapsidated in the coat protein of helper begomoviruses and explains the wide-spread transmission of CLCuMuB [43].

**Figure 2:**
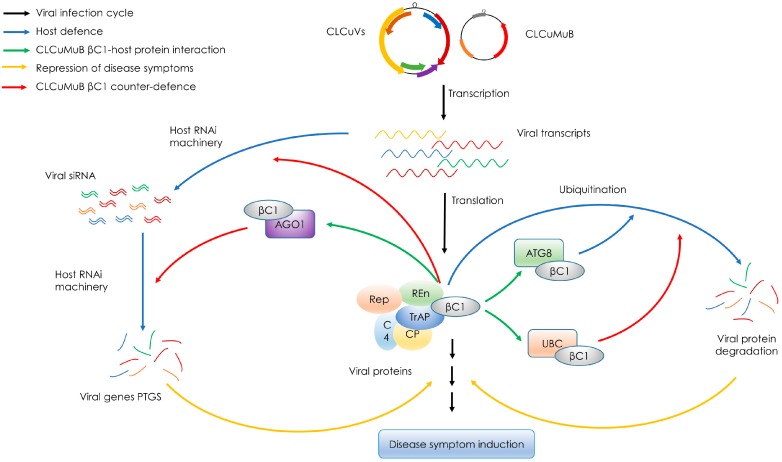
A working model for the role of *Cotton leaf curl Multan betasatellite* (CLCuMuB) βC1 protein in disease progression and suppression of host defense. Black arrows indicate un-interrupted virus infection cycle, blue arrows represent host defense mechanisms, green arrows represent βC1 interaction with host proteins, orange arrows represent the repression of leaf curl disease symptoms and red arrows represent the counter-defense mechanisms triggered by CLCuMuB βC1. Begomoviruses associated with cotton leaf curl disease (CLCuVs), along with CLCuMuB, produce transcripts of virus genes and *βC1*, respectively. In an un-interrupted disease infection cycle, these transcripts are translated in respective proteins, that in-turn interact with several cellular proteins that induces typical leaf curl disease symptoms. But usually the viral transcripts are targeted by the host’s RNA interference (RNAi) machinery that subsequently degrades the viral gene transcripts in a process called post-transcriptional gene silencing (PTGS) and interferes with disease progression and symptom development. CLCuMuB βC1 has been reported to interact with host protein AGO1 and acts as a strong suppressor of PTGS; this assisting the helper virus in normal disease progression and symptom development. CLCuMuB βC1 can also suppress systemic gene silencing, resulting in reduced levels of viral siRNAs. Viral proteins and βC1 are also targeted by cellular autophagy machinery that degrades these proteins through a cellular mechanism called ubiquitination. Host protein ATG8 has been reported to interact with CLCuMuB βC1 in an attempt to induce autophagy. CLCuMuB βC1 has also been reported to interact with a cellular protein UBC, and this interaction interferes with the ubiquitination, leading the infected cells towards disease progression and symptom development. It must be noted that only the functional roles of CLCuMuB βC1, and among those only the ones that are experimentally confirmed, have been shown here; the functions and βC1-host protein interaction may vary for different species of betasatellites.

Begomoviruses are targeted by host RNA interference (RNAi) machinery, a host antiviral defense mechanism that results in post-transcriptional gene silencing (PTGS) of viral transcripts. As a counter-defense mechanism, begomovirus/betasatellite proteins act as suppressors of PTGS. After infection, the virus derived small interfering RNA (siRNA) accumulate in host cells, and the level of these accumulated siRNAs are negatively correlated with symptoms severity [44,45]. CLCuMuB βC1 can suppress host defense system by functioning as a strong suppressor of gene silencing [46] (Figure 2). CLCuMuB βC1 can also suppress systemic gene silencing, resulting in reduced levels of viral siRNAs [47]. CLCuMuB βC1 has been demonstrated to physically interact, in yeast-two hybrid system, with protein argonaute-1 (AGO1; Figure 2), an important component of host RNAi pathway that binds with the siRNAs and represses the translation of respective RNAs [48]. The suppression of PTGS by βC1 can be correlated with the strong role of CLCuMuB in CLCuD pathogenicity and symptom development [46].

CLCuMuB βC1 has been identified to strongly interact with autophagy-related ATG8 protein [49] (Figure 2), a ubiquitin-like protein that is involved in cargo recruitment into phagophores and the biogenesis of autophagosomes [50]. The induction of autophagy by begomovirus (CLCuMuV) and a strong ATG8-βC1 interaction might indicate that autophagy functions as an antiviral mechanism against CLCuMuV by degrading βC1 via its recruitment to autophagosomes through ATG8-related proteins [49]. βC1 protein of CLCuMuB has also been shown to interact with a tomato ubiquitin-conjugating enzyme (UBC) [51] (Figure 2), an enzyme that has an important role in protein degradation. UBC generates poly-ubiquitin chains that link to the substrates for recognition by the 26S proteasome, and ultimately assist substrate degradation [52]. It is suggested that βC1-UBC interaction interferes with UBC activity, leading to the interference in the ubiquitin proteasome pathway, to increase accumulation of βC1 protein; this might lead to typical leaf curl disease symptom development in infected plants [51]. Moreover, protein-protein interaction studies have also revealed a potential interaction of CLCuMuB βC1 with host proteins that participate in metabolic and defense pathways for example retinoblastoma-related protein, ABC-transporter proteins, receptor kinases, salicylic acid, carboxyl methyltransferase, and ubiquitin-conjugating enzyme [53]; suggesting the pivotal role of CLCuMuB in CLCuD development and progression.

## 4. Trans-Replication of Cotton Leaf Curl Multan Betasatellite and Its Association with Bipartite Tomato Leaf Curl New Delhi Virus

Betasatellites do not encode their own replication protein and must be *trans*-replicated by the Rep encoded by the helper viruses. The helper begomovirus mainly replicate via rolling circle replication (RCR) mechanism by using double stranded (ds) DNA intermediate during replication [54]. Betasatellites also use dsDNA intermediate for DNA replication similar to begomoviruses [55]. Analysis of CLCuMuB DNA intermediates, formed during *trans*-replication by helper virus, suggests that CLCuMuB and its helper virus employ similar strategies for DNA replication [55]. However, unlike DNA-B of bipartite begomoviruses, where replication depends on the specific Rep of cognate DNA-A, CLCuMuB can be *trans*-replicated by diverse begomoviruses [56]. CLCuMuB is reported to infect cotton with *Okra enation leaf curl virus* [57] and ToLCNDV [4]. CLCuMuB has also been reported to infect non-cotton hosts like tomato (*Solanum lycopersicum*), bitter gourd (*Momordica* spp.) and luffa (*Luffa* spp.) with the helper ToLCNDV, in chili (*Capsicum annuum*) with *Bhendi yellow vein mosaic virus* [58], in *Sonchus arvensis* with *Alternanthera yellow vein virus* [59], in sunn hemp (*Crotalaria juncea*) with CLCuKoV-Bur [60], in China rose (*Hibiscus rosa*-*sinensis*) with CLCuKoV [61], and in cluster bean (*Cyamopsis tetragonoloba*) with PaLCuV [62]. Under experimental conditions, CLCuMuB produced mild symptoms in cotton when bombarded with *Tomato leaf curl virus* (ToLCV) [63]; and in the presence of CLCuMuB, the symptoms of a NW begomovirus *Cabbage leaf curl virus* (CbLCuV) were also enhanced in *Nicotiana benthamiana* [10].

CLCuMuB was initially found to be associated with monopartite begomoviruses in the OW, but later results showed that CLCuMuB interacts with DNA-A of bipartite begomoviruses under experimental conditions and are also found associated with some bipartite begomoviruses, like ToLCNDV, in field conditions [64,65,66]. The accumulation of ToLCNDV DNA-A and ToLCNDV DNA-B increased to several fold when co-inoculated with CLCuMuB [66]. Interestingly, CLCuMuB substitutes the role of DNA-B of a bipartite begomovirus ToLCNDV upon systemic infection in host plants [42,67]. The Rep of bipartite begomoviruses is known to associate specifically with cognate DNA-B [54,68,69]. The promiscuous replicative nature of betasatellites suggests that recognition between Rep and betasatellites are more flexible, in comparison to species-specific replication of bipartite begomoviruses DNA-A and DNA-B [56]. The interaction between CP of helper virus and βC1 could possibly control intracellular movement of helper virus [70]. However, a recent study has shown that CLCuMuB localizes only in the phloem tissues of host and, unlike the DNA-B component, it is unable to release the monopartite helper virus out of phloem tissues [71]. Taken together, the wide range of CLCuMuB *trans*-replication ability is probably the main reason of its widespread pathogenicity and rapid evolution.

## 5. Recombination and Phylogeny of Cotton Leaf Curl Multan *Betasatellite*

**Figure 3:**
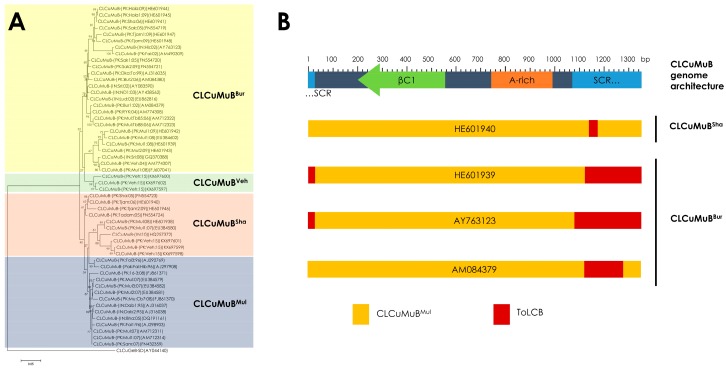
Phylogeny and recombination of *Cotton leaf curl Multan Betasatellite* (CLCuMuB). (**A**) Phylogenetic dendrogram has been shown to indicate four strains of CLCuMuB; Burewala strain (CLCuMuB^Bur^), Vehari strain (CLCUMuB^Veh^), Shahdadpur strain (CLCuMuB^Sha^), and Multan Strain (CLCuMuB^Mul^). The phylogenetic tree is based on the information from Akhtar et al. [39] and Zubair et al. [36]. (**B**) The recombination pattern of CLCuMuB. Genomic architecture of CLCuMuB has been described with coordinates of satellite conserved region (SCR), adenine-rich region (A-rich), and *βC1* gene. The recombinant strains of CLCuMuB (CLCuMuB^Bur^ and CLCuMuB^Sha^) have been shown with representative accessions. CLCuMuB^Bur^ contains a recombinant sequence within the SCR derived from a distinct betasatellite, *Tomato leaf curl betasatellite* (ToLCB). CLCuMuB^Sha^ also contains a smaller recombinant fragment within the SCR derived from ToLCB. The recombination pattern is inspired from Akhtar et al. [39].

Based on phylogeny and recombination, CLCuMuB is classified into four strains (Figure 3A), Multan strain (CLCuMuB^Mul^), Burewala strain (CLCuMuB^Bur^), Shahdadpur strain (CLCuMuB^Sha^) and Vehari strain (CLCuMuB^Veh^) [36,39]. The first epidemic of CLCuD in Pakistan during the 1990s was associated with a single strain CLCuMuB^Mul^, with multiple monopartite begomoviruses [22,27,28]. The strain of CLCuMuB associated with the resistance breaking CLCuKoV-Bur during the second epidemic of CLCuD in Pakistan in the early 2000s was CLCuMuB^Bur^. This strain was a recombinant, with a small replacement of sequences within the SCR derived from a distinct betasatellite, *Tomato leaf curl betasatellite* (ToLCB; Figure 3B) [30,31]. In 2005, an increase in CLCuD in Sindh province of Pakistan was shown to be associated with a new strain of CLCuMB containing a smaller recombinant fragment from ToLCB, later referred to as the CLCuMuB^Sha^ (Figure 3B) [30]. In 2015, a new recombinant strain, CLCuMuB^Veh^, was associated with begomovirus disease complex identified from Pakistan in association with multiple monopartite begomoviruses that were predominant in 1990s [36]. It must be noted that in all the recombinant CLCuMuB strains, recombination occurred in SCR (Figure 3B) [36,39]. This might be linked to the high variability between the SCR and the A-rich region of CLCuMuB [31]. On the other hand, analysis of the predicted amino acid sequences of the βC1 has shown that it remains highly conserved [39], which is plausible given the important role of βC1 in pathogenicity and host interaction (explained in Section 3). To date, more than 200 full-length nucleotide sequences of CLCuMuB genomes have been isolated from cotton, sequenced, and submitted in the nucleotide database (Table S1).

CLCuMuB associated with CLCuD complex showed significant changes post-resistance breaking, specifically recombination within the SCR [31]. The SCR region has been considered as a hotspot for recombination in begomovirus disease complexes. The position of SCR region in betasatellites is parallel to the position of common region (CR) in bipartite begomoviruses. The CR in intergenic region of bipartite begomoviruses is part of a promoter which initiates complementary-sense gene expression. The SCR region is located in intergenic region of betasatellites but does not contain promoter elements. According to current model of begomovirus replication, Rep binds to specific iterative sequences (iterons) located upstream of potential stem-loop structure. The nonanucleotide sequence within hairpin-loop structure near to SCR region contains nicking site, which is essential for initiation of begomovirus and betasatellite replication [54]. It was hypothesized that SCR region immediately upstream of the stem-loop and the nicking site, likely to be involved in betasatellite replication. All defective betasatellites identified so far have maintained their SCRs, which further supports this hypothesis [40,72,73,74]. However, betasatellites (including CLCuMuB [39] and *Ageratum yellow vein betasatellite* [75]) lack iterons; they rather contain iteron-like sequences, that appear to be situated in the sequence between the SCR and the A-rich region [76]. This suggests that Rep recognizes the origin of replication in betasatellites by different mechanism than the bipartite begomoviruses [74,77]. Taken together, recombination has a major role in the evolution of CLCuMuB and SCR within CLCuMuB is probably the hotspot for recombination.

## 6. Conclusions and Future Prospects

CLCuD is an important disease and a serious threat for cotton production in the Indian subcontinent. A major pathogenicity determinant of CLCuD is CLCuMuB, which is widely associated with begomoviruses causing CLCuD. βC1, a single protein produced by CLCuMuB, is a multifunctional protein and plays a critical role in (1) suppression of RNAi-mediated host defense, (2) suppression of ubiquitination and protein degradation, that results in elevating βC1 accumulation and assisting the leaf curl disease severity, and (3) interacting with several host proteins that are involved in defense and metabolic pathways, ultimately assisting the helper virus to hijack the cellular machinery and spread of the disease. CLCuMuB in the OW, like its helper viruses, is evolving rapidly through recombination and the SCR region of CLCuMuB appears to be the hotspot for recombination. However, the precise role of CLCuMuB SCR remains unknown. Future experiments may shed light on the SCR as recombination hotspot in betasatellites, and specifically CLCuMuB. The steps in the RNA silencing pathway that are targeted by the CLCuMuB βC1 protein also remain to be elucidated. Moreover, the interaction of betasatellites and βC1 with the proteome of vector *B. tabaci* is poorly understood and remains to be explored. Apart from basic understanding, further research on CLCuMuB has great potential for practical applications, for example in the rapidly boosting field of plant genome editing. Several geminiviruses have been demonstrated as efficient vectors to deliver reagents for plant genome editing [78,79,80]; where CLCuMuB has been utilized as a vector for gene delivery in multiple studies [81,82], the efficacy of CLCuMuB as a vector for plant genome editing remains to be evaluated [78]. An efficient genome editing tool, clustered regularly interspaced short palindromic repeats/CRISPR-associated9 (CRISPR/Cas9), has been demonstrated to engineer resistance against several geminiviruses [83,84,85], including CLCuKoV [86], by targeting and cleaving the genome of invading geminiviruses [87,88,89]. Where resistance to CLCuD [90] in transgenic tobacco plants expressing intron-hairpin RNA from CLCuMuB βC1 [91] and transgenic cotton plants expresing hairpin RNA from βC1-AC1 (AC1 of CLCuKoV-Bu) [92] has been demonstrated, the utility of the CRISPR/Cas9 system, and Cas9 variants like Cpf1 [93,94], to target CLCuMuB in effort to engineer CLCuD resistance remains to be evaluated.

## Supplementary Materials

The following are available online at www.mdpi.com/1999-4915/9/10/280/s1, Table S1: List of *Cotton leaf curl Multan betasatellite* accessions identified from cotton.
