# Supplementary material for: An Insight into Cotton Leaf Curl Multan Betasatellite, the Most Important Component of Cotton Leaf Curl Disease Complex
Source: Viruses. 2017 Sep 29;9(10):280. doi: 10.3390/v9100280 (PMC5691632; doi:10.3390/v9100280)
Supplement: Supplementary file 1 [file viruses-09-00280-s001.pdf]

Supplementary Information

Table S1: List of *Cotton leaf curl Multan betasatellite* accessions identified from cotton

No.	Host	Year	Location	Accession numbers
1	*Gossypium hirsutum*	1996	Pakistan	AJ298903
2	*Gossypium hirsutum*	1995	India	AJ291601
3	*Gossypium hirsutum*	1996	Sudan	AY077797
4	*Gossypium hirsutum*	2000	Pakistan	AJ292769
5	*Gossypium hirsutum*	2001	India	AJ316037
6	*Gossypium hirsutum*	2001	India	AJ316038
7	*Gossypium hirsutum*	2001	Sudan	AY044140
10	*Gossypium hirsutum*	2001	India	AJ315700
11	*Gossypium hirsutum*	2004	Sudan	AY669328
12	*Gossypium hirsutum*	2004	India	AY705381
13	*Gossypium hirsutum*	2004	India	NC_007219
14	*Gossypium hirsutum*	2005	India	DQ191161
15	*Gossypium hirsutum*	2005	Sudan	NC_006935
16	*Gossypium hirsutum*	2006	India	DQ364230
17	*Gossypium punctatum*	2006	Pakistan	EU384588
18	*Gossypium punctatum*	2006	Pakistan	EU384580
19	*Gossypium punctatum*	2006	Pakistan	EU384581
20	*Gossypium punctatum*	2006	Pakistan	EU384582
21	*Gossypium punctatum*	2006	Pakistan	EU384583
22	*Gossypium punctatum*	2006	Pakistan	EU384584
23	*Gossypium punctatum*	2006	Pakistan	EU384585
24	*Gossypium punctatum*	2006	Pakistan	EU384586
25	*Gossypium punctatum*	2006	Pakistan	EU384588
26	*Gossypium punctatum*	2006	Pakistan	EU384589
27	*Gossypium latifolium*	2006	Pakistan	EU384590
28	*Gossypium davidsonii*	2006	Pakistan	EU384592
29	*Gossypium darwinii*	2006	Pakistan	EU386596
30	*Gossypium darwinii*	2006	Pakistan	EU386597
31	*Gossypium darwinii*	2006	Pakistan	EU386598
32	*Gossypium darwinii*	2006	Pakistan	EU386599
33	*Gossypium darwinii*	2006	Pakistan	EU386600
34	*Gossypium darwinii*	2006	Pakistan	EU386601
35	*Gossypium darwinii*	2006	Pakistan	EU386602
36	*Gossypium darwinii*	2006	Pakistan	EU386603
37	*Gossypium darwinii*	2006	Pakistan	EU386604
38	*Gossypium darwinii*	2006	Pakistan	EU384605

39	*Gossypium punctatum*	2006	Pakistan	EU384587
40	*Gossypium hirsutum*	2007	Pakistan	AM774306
41	*Gossypium hirsutum*	2007	Pakistan	AM774307
42	*Gossypium hirsutum*	2007	Pakistan	AM774308
43	*Gossypium hirsutum*	2007	Pakistan	AM774309
44	*Gossypium hirsutum*	2007	Pakistan	AM774310
45	*Gossypium hirsutum*	2007	Pakistan	AM774311
46	*Gossypium hirsutum*	2007	Pakistan	AM774312
47	*Gossypium hirsutum*	2007	Pakistan	AM774313
48	*Gossypium hirsutum*	2008	India	GQ369730
49	*Gossypium hirsutum*	2008	India	GQ369730
50	*Gossypium hirsutum*	2008	India	GQ369730
51	*Gossypium hirsutum*	2009	India	NC_013802
52	*Gossypium hirsutum*	2009	India	FN658722
53	*Gossypium hirsutum*	2009	India	FN658737
54	*Gossypium hirsutum*	2009	Pakistan	FN554719
55	*Gossypium hirsutum*	2009	Pakistan	FN554720
56	*Gossypium hirsutum*	2009	Pakistan	FN554721
57	*Gossypium hirsutum*	2009	Pakistan	FN554722
58	*Gossypium hirsutum*	2009	Pakistan	FN554723
59	*Gossypium hirsutum*	2009	Pakistan	FN554724
60	*Gossypium hirsutum*	2010	India	GU440581
61	*Gossypium hirsutum*	2010	China	GQ906588
62	*Gossypium hirsutum*	2010	India	HM461864
63	*Gossypium hirsutum*	2010	India	HM461865
64	*Gossypium hirsutum*	2010	India	HM037921
65	*Gossypium hirsutum*	2010	India	HM037922
66	*Gossypium hirsutum*	2010	India	HM146307
67	*Gossypium hirsutum*	2010	India	HM146308
68	*Gossypium hirsutum*	2010	India	HQ158008
69	*Gossypium hirsutum*	2010	India	HQ343201
70	*Gossypium hirsutum*	2010	Pakistan	HF542911
71	*Gossypium hirsutum*	2010	India	NC_017829
72	*Gossypium hirsutum*	2010	India	FR751147
73	*Gossypium hirsutum*	2010	India	FR751148
74	*Gossypium hirsutum*	2011	India	JF502374
75	*Gossypium hirsutum*	2011	India	JF502375
76	*Gossypium hirsutum*	2011	India	JF502376
77	*Gossypium hirsutum*	2011	India	JF502377
78	*Gossypium hirsutum*	2011	India	JF502378
79	*Gossypium hirsutum*	2011	India	JF502379
80	*Gossypium hirsutum*	2011	India	JF502380

81	*Gossypium hirsutum*	2011	India	JF502381
82	*Gossypium hirsutum*	2011	India	JF502382
83	*Gossypium hirsutum*	2011	India	JF502383
84	*Gossypium hirsutum*	2011	India	JF502384
85	*Gossypium hirsutum*	2011	India	JF502385
86	*Gossypium hirsutum*	2011	India	JF502386
87	*Gossypium hirsutum*	2011	India	JF502387
88	*Gossypium hirsutum*	2011	India	JF502388
89	*Gossypium hirsutum*	2011	India	JF502389
90	*Gossypium hirsutum*	2011	India	JF502390
91	*Gossypium hirsutum*	2011	India	JF502391
92	*Gossypium hirsutum*	2011	India	JF502392
93	*Gossypium hirsutum*	2011	India	JF502393
94	*Gossypium hirsutum*	2011	India	JF502394
95	*Gossypium hirsutum*	2011	India	JF502395
96	*Gossypium hirsutum*	2011	India	JF502396
97	*Gossypium hirsutum*	2011	India	JF502397
98	*Gossypium hirsutum*	2011	India	JF502398
99	*Gossypium hirsutum*	2011	India	JF509751
100	*Gossypium hirsutum*	2011	India	JF509752
101	*Gossypium hirsutum*	2011	China	JQ317604
102	*Gossypium hirsutum*	2011	India	FR877537
103	*Gossypium hirsutum*	2011	Pakistan	HE601938
104	*Gossypium hirsutum*	2011	Pakistan	HE601939
105	*Gossypium hirsutum*	2011	Pakistan	HE601940
106	*Gossypium hirsutum*	2011	Pakistan	HE601941
107	*Gossypium hirsutum*	2011	Pakistan	HE601942
108	*Gossypium hirsutum*	2011	Pakistan	HE601943
109	*Gossypium hirsutum*	2011	Pakistan	HE601944
110	*Gossypium hirsutum*	2011	Pakistan	HE601945
111	*Gossypium hirsutum*	2011	Pakistan	HE601946
112	*Gossypium hirsutum*	2011	Pakistan	HE601947
113	*Gossypium hirsutum*	2011	Pakistan	HE601948
114	*Gossypium hirsutum*	2012	Pakistan	HF549185
115	*Gossypium hirsutum*	2012	Pakistan	HF549186
116	*Gossypium hirsutum*	2012	Pakistan	HF549187
117	*Gossypium hirsutum*	2012	Pakistan	HF549188
118	*Gossypium hirsutum*	2012	China	KC171655
119	*Gossypium hirsutum*	2012	Pakistan	HE965435
120	*Gossypium hirsutum*	2012	Pakistan	HE965436
121	*Gossypium hirsutum*	2012	Pakistan	HE965437
122	*Gossypium hirsutum*	2012	Pakistan	HE965438

123	*Gossypium hirsutum*	2012	Pakistan	HE965439
124	*Gossypium hirsutum*	2012	Pakistan	HE965440
125	*Gossypium hirsutum*	2012	Pakistan	HE965441
126	*Gossypium hirsutum*	2012	Pakistan	HE965442
127	*Gossypium hirsutum*	2012	Pakistan	HE965443
128	*Gossypium hirsutum*	2012	Pakistan	HE965444
129	*Gossypium hirsutum*	2012	Pakistan	HE965445
130	*Gossypium hirsutum*	2012	Pakistan	HE965446
131	*Gossypium hirsutum*	2012	Pakistan	HE965447
132	*Gossypium hirsutum*	2012	Pakistan	HE965448
133	*Gossypium hirsutum*	2012	Pakistan	HE965449
134	*Gossypium hirsutum*	2012	Pakistan	HE965450
135	*Gossypium hirsutum*	2012	Pakistan	HE965451
136	*Gossypium hirsutum*	2012	Pakistan	HE965452
137	*Gossypium hirsutum*	2012	Pakistan	HE965453
138	*Gossypium hirsutum*	2012	Pakistan	HE965454
139	*Gossypium hirsutum*	2012	Pakistan	HE965455
140	*Gossypium hirsutum*	2012	Pakistan	HE965456
141	*Gossypium hirsutum*	2012	Pakistan	HE978339
142	*Gossypium hirsutum*	2012	Pakistan	HE978340
143	*Gossypium hirsutum*	2012	Pakistan	HE978341
144	*Gossypium hirsutum*	2012	Pakistan	HE978342
145	*Gossypium hirsutum*	2012	Pakistan	HE978343
146	*Gossypium hirsutum*	2012	Pakistan	HE978344
147	*Gossypium hirsutum*	2012	Pakistan	HF564596
148	*Gossypium hirsutum*	2012	Pakistan	HF564596
149	*Gossypium hirsutum*	2012	Pakistan	HF564597
150	*Gossypium hirsutum*	2012	Pakistan	HF564598
151	*Gossypium hirsutum*	2012	Pakistan	HF564599
152	*Gossypium hirsutum*	2012	Pakistan	HF564603
153	*Gossypium hirsutum*	2012	Pakistan	HF564604
154	*Gossypium arboreum*	2013	Pakistan	HF912231
155	*Gossypium arboreum*	2013	Pakistan	HF912232
156	*Gossypium hirsutum*	2013	Pakistan	HF952152
157	*Gossypium hirsutum*	2013	Pakistan	HF952153
158	*Gossypium hirsutum*	2013	Pakistan	HF952156
159	*Gossypium arboreum*	2013	Pakistan	HG428700
160	*Gossypium hirsutum*	2013	Pakistan	HG428701
161	*Gossypium hirsutum*	2013	Pakistan	HG428702
162	*Gossypium hirsutum*	2013	Pakistan	HG428703
163	*Gossypium hirsutum*	2013	Pakistan	HG428707
164	*Gossypium hirsutum*	2013	Pakistan	HG422577

165	*Gossypium hirsutum*	2013	Pakistan	HG422578
166	*Gossypium hirsutum*	2013	Pakistan	HG422579
167	*Gossypium hirsutum*	2013	Pakistan	HG422580
168	*Gossypium hirsutum*	2013	Pakistan	HG422581
169	*Gossypium hirsutum*	2013	Pakistan	HG422582
170	*Gossypium hirsutum*	2013	Pakistan	HG422583
171	*Gossypium hirsutum*	2013	Pakistan	HG000665
172	*Gossypium hirsutum*	2014	India	KP015741
173	*Gossypium hirsutum*	2014	India	KP015742
174	*Gossypium hirsutum*	2014	India	KP015743
175	*Gossypium hirsutum*	2014	Pakistan	HG934394
176	*Gossypium hirsutum*	2014	Pakistan	HG934395
177	*Gossypium hirsutum*	2014	Pakistan	HG934396
178	*Gossypium hirsutum*	2014	Pakistan	LK995398
179	*Gossypium hirsutum*	2014	Pakistan	LK995399
180	*Gossypium hirsutum*	2014	Pakistan	HF567946
181	*Gossypium hirsutum*	2014	India	KJ614434
182	*Gossypium hirsutum*	2014	India	KJ614435
183	*Gossypium hirsutum*	2014	India	KJ614436
184	*Gossypium hirsutum*	2014	India	KM070822
185	*Gossypium hirsutum*	2015	China	KP762787
186	*Gossypium hirsutum*	2015	Pakistan	KR816003
187	*Gossypium hirsutum*	2015	Pakistan	KR816004
188	*Gossypium hirsutum*	2015	Pakistan	KR816005
189	*Gossypium hirsutum*	2015	Pakistan	KR816006
190	*Gossypium hirsutum*	2015	Pakistan	KR816007
191	*Gossypium hirsutum*	2015	India	KT228323
192	*Gossypium hirsutum*	2015	India	KT228324
193	*Gossypium hirsutum*	2015	India	KT228325
194	*Gossypium hirsutum*	2015	India	KT228326
195	*Gossypium hirsutum*	2015	Pakistan	LN845926
196	*Gossypium hirsutum*	2015	Pakistan	LN845927
197	*Gossypium hirsutum*	2015	Pakistan	LN845928
198	*Gossypium hirsutum*	2015	Pakistan	LN867444
199	*Gossypium hirsutum*	2015	Pakistan	LN867445
200	*Gossypium hirsutum*	2015	Pakistan	LN867446
201	*Gossypium hirsutum*	2015	Pakistan	LN867447
202	*Gossypium hirsutum*	2015	Pakistan	LN867448
203	*Gossypium hirsutum*	2015	Pakistan	LN867449
204	*Gossypium hirsutum*	2015	Pakistan	LN867450
205	*Gossypium hirsutum*	2015	Pakistan	LN908793
206	*Gossypium hirsutum*	2015	Pakistan	LN908794

207	*Gossypium hirsutum*	2016	Pakistan	KX656816
208	*Gossypium hirsutum*	2016	Pakistan	KX656817
209	*Gossypium hirsutum*	2016	Pakistan	KX656818
210	*Gossypium hirsutum*	2016	Pakistan	KX656819
211	*Gossypium hirsutum*	2016	Pakistan	KX656820
212	*Gossypium hirsutum*	2016	Pakistan	KX656821
213	*Gossypium hirsutum*	2016	Pakistan	KX656822
214	*Gossypium hirsutum*	2016	Pakistan	KX656823
215	*Gossypium hirsutum*	2016	Pakistan	KX656824
216	*Gossypium hirsutum*	2016	Pakistan	KX656825
217	*Gossypium hirsutum*	2016	Pakistan	KX656826
218	*Gossypium hirsutum*	2016	Pakistan	KX656827
219	*Gossypium hirsutum*	2016	Pakistan	KX656828
